# A Sponsor’s Best Practice and Operating Principles to Manage Data Monitoring Committees

**DOI:** 10.1007/s43441-025-00742-w

**Published:** 2025-01-07

**Authors:** Malene Muusfeldt Birck, Josephine Skovgaard Rasmussen, Ida Carøe Helmark, Karsten Lollike

**Affiliations:** 1 Safety Surveillance, Novo Nordisk A/S, Copenhagen, Denmark; 2 Medical & Science, Novo Nordisk A/S, Copenhagen, Denmark; 3 Global Patient Safety, Novo Nordisk A/S, Copenhagen, Søborg, 2860 Denmark

**Keywords:** Data Monitoring Committee, Data Safety Monitoring Board, Operating principles, Regulatory requirements, Independency, Data quality

## Abstract

The use of data monitoring committees (DMC) to safeguard patients’ safety in clinical trials has evolved over the last decades and has become increasingly common. To ensure well-operating and high-performing DMCs, pharmaceutical companies need to establish clearly defined operational processes while continuously seeking to optimize these and adapt to the needs of drug development. Although there are health authority guidelines on establishing and managing a DMC, the perspectives and experiences of sponsors are often underrepresented. This publication shares insights on a sponsor, Novo Nordisk (NN), regarding principles and practices for DMC establishment and management across varying trial types and therapeutic areas, including challenges and solutions. Highlighting NN’s structured and successful approach to DMCs, it details clearly defined roles and responsibilities that ensure productive DMC meetings and high-quality data for the DMC. Additionally, NN’s practices for clear, transparent, and trustful communication between the sponsor, the DMC, and the independent external statistical vendor are described. Processes for quality control, internal audits, and learnings from inspections and how these are incorporated for continuous improvement of the DMC process are discussed. While the processes and practices described are primarily designed for medium and large pharmaceutical companies, certain aspects may also be relevant and beneficial for smaller companies.

## Introduction

**Fig. 1:**
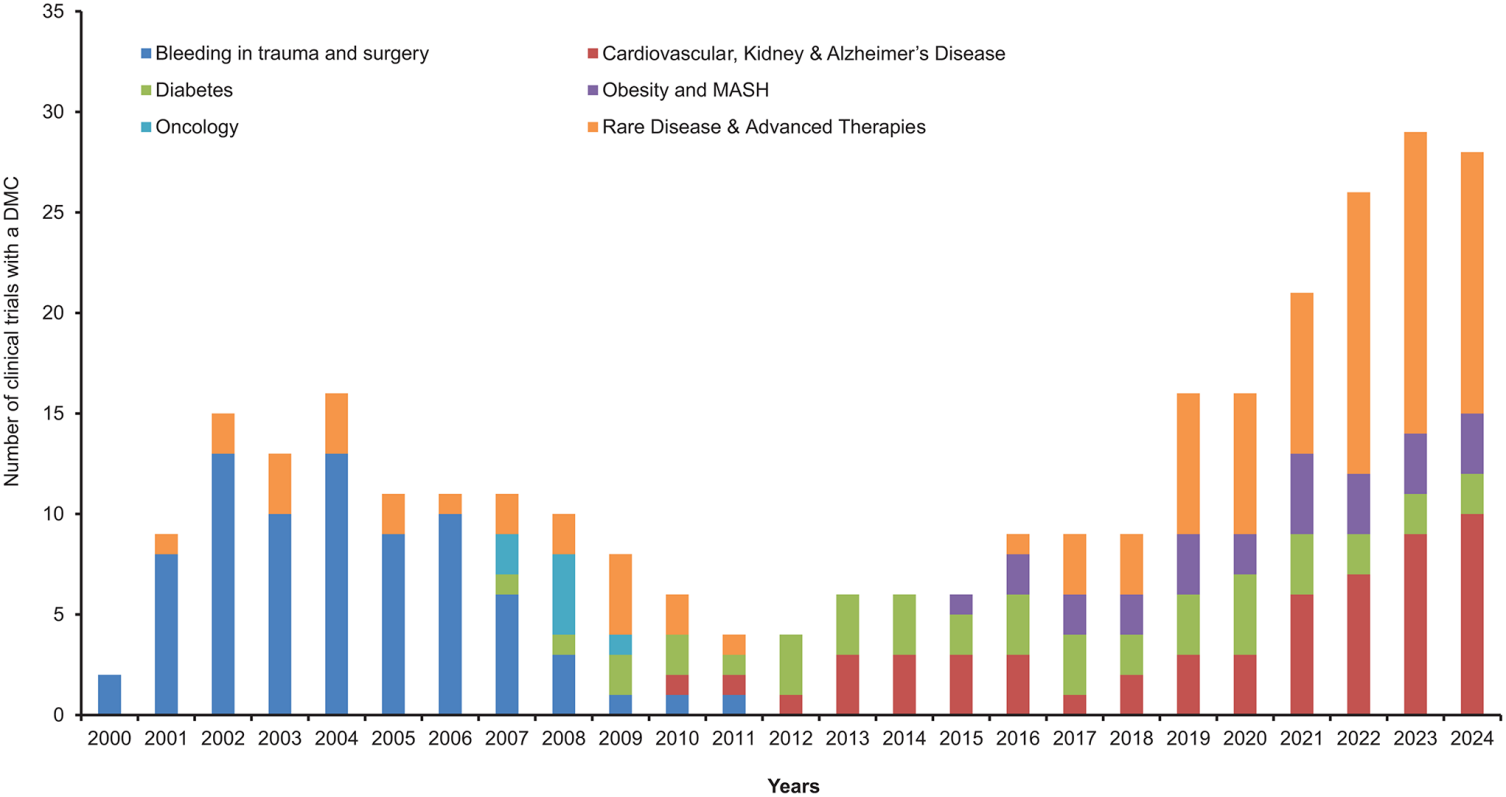
Novo Nordisk clinical trials with established data monitoring committee. Abbreviation: Metabolic dysfunction-associated steatohepatitis (MASH)

For many clinical trials, a Data Monitoring Committee (DMC) or Data Safety Monitoring Board (DSMB) is a critical component of maintaining clinical trial oversight [1–3]. Historically, sponsor-appointed DMCs have mostly been established to monitor large, late-stage trials with major mortality-morbidity endpoints and trials involving vulnerable populations. Over the past decades, the use of DMCs in clinical drug development has evolved with increasingly complex trial designs and regulatory expectations, extending beyond large trials involving serious morbidity or mortality [4, 5]. A single DMC may monitor multiple trials within a development program [6–8] and may be responsible for interim efficacy analyses to provide recommendations to the sponsor for early trial termination [9–11]. Over the years, Novo Nordisk (NN) has expanded into novel therapeutic areas, and the number of DMCs established for NN-sponsored trials has risen significantly (Fig. 1).

**Fig. 2:**
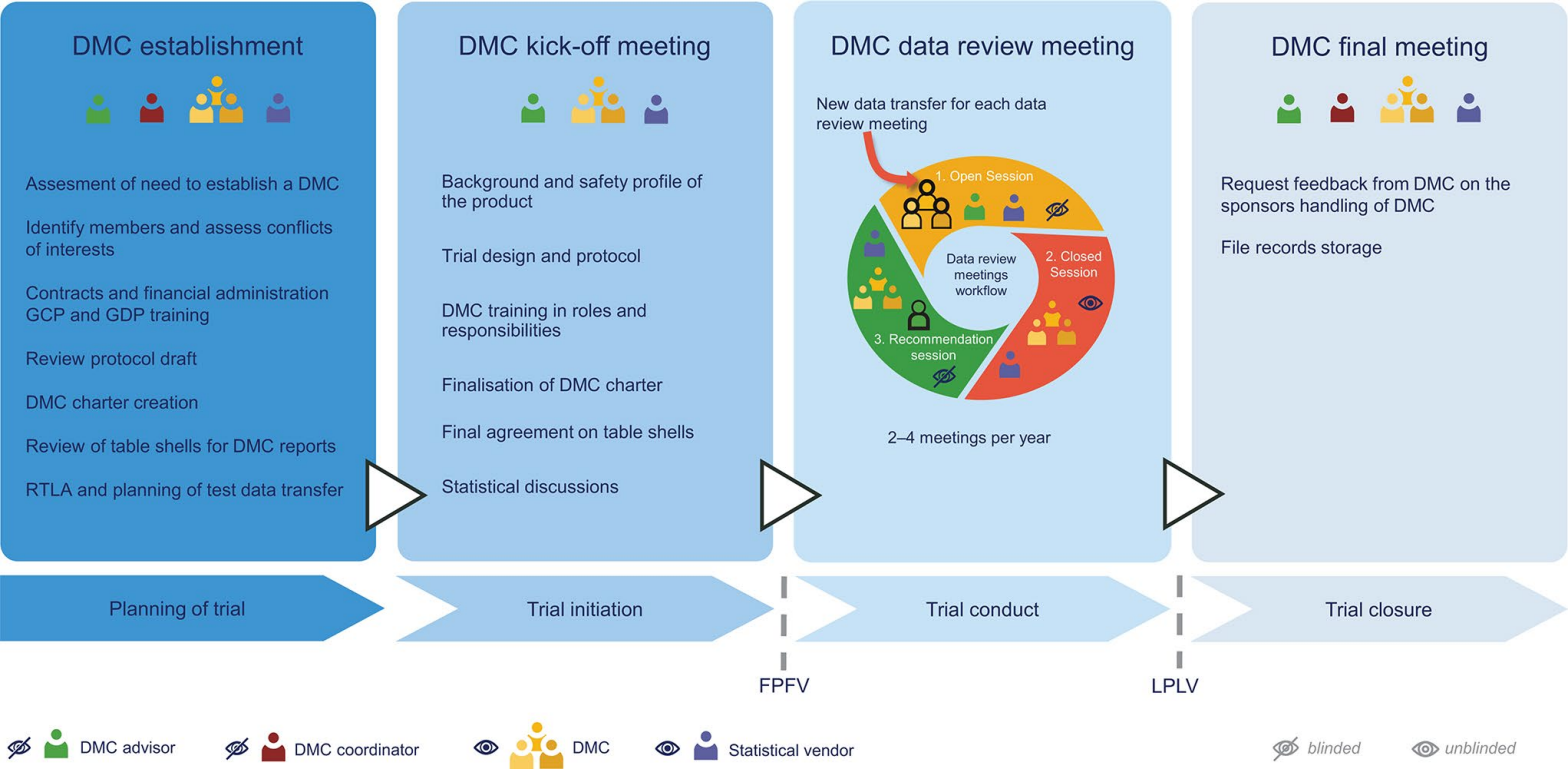
Novo Nordisk data monitoring committee process overview. Abbreviations: Data Monitoring Committee (DMC); Good Clinical Practice (GCP); Good Documentation Practice (GDP); Responsibility, Timelines and Logistics Agreement (RTLA); First Patient First Visit (FPFV); Last Patient Last Visit (LPLV)

Guidelines and publications exist discussing various perspectives on using a DMC; however, sponsors’ experiences and perspectives remain underrepresented [3, 4, 12, 13]. This publication shares insights into NN processes for in-house handling and management of well-operating and high-performing DMCs by ensuring high-quality data for DMC data reports and planning productive and effective DMC meetings. This is achieved and maintained by applying a structured process, stringent quality control, clearly defined roles and responsibilities, and transparent communication between the sponsor, the DMC and the independent external statistical vendor (Fig. 2). With decades of experience in pharmacovigilance and drug development, our aim with this publication is to share a sponsor’s perspectives on processes, operating principles, and recommendations for good practice in establishing and managing DMCs across different clinical trials and therapeutical areas, including challenges faced.

## Establishment of a DMC

Sponsors may have different areas to drive the formation and facilitate the DMC work. At NN, DMCs are overseen by the Pharmacovigilance area. A Global Patient Safety Surveillance department representative, hereafter termed ‘DMC advisor’, plays a key role in DMC management and serves as the central point of contact for the DMC. The DMC advisor drives the sponsor assessment for whether a DMC is required and, subsequently, the DMC composition and expertise areas to be included.

The proposal for a DMC is submitted to the internal NN safety committee, which consists of representatives from various functional areas. This committee is the sole body responsible for evaluating the product’s safety profile, benefit-risk balance, and communications related to product safety. The safety committee supports the DMC proposal and considers whether it is advantageous to have a single DMC oversee multiple related trials. Having one DMC monitor several trials within the development program allows for synergy and provides the committee with more data to make informed assessments and recommendations. These decisions should ideally be made early in the trial planning process, since it takes time to identify and contract DMC members and establish the committee.

Typically, DMCs for NN sponsored trials consist of three to six members, including a DMC statistician and a DMC chair, in accordance with guidelines [3, 14, 15]. It is crucial that DMC members are independent of the sponsor, meaning that they cannot be an investigator in one of the sponsor’s trials or part of related committees such as steering or event adjudication committees, and advisory boards. To preclude bias in data assessment, DMC members are prohibited from having any financial, intellectual, or emotional competing interests such as serving on a competitor’s DMC, i.e. within the same indication and the same mode of action or serving on a competitor advisory board. These requirements are contractually binding and detailed in the DMC charter.

When a candidate for a DMC has been identified and cleared from conflicts of interest (COI), the following is obtained: a signed General Data Protection Regulation (GDPR) consent form to comply with EU data legislation, a current curriculum vitae, and documentation of training in Good Clinical Practice. Once a DMC member is contracted, they are subsequently blocked from NN expert directories, preventing members from being approached for other sponsor-related activities during their DMC service. All DMC members are reminded in relation to the data review meetings to disclose any new obligations after contract signing to assess potential COI.

The DMC should be established in parallel with protocol development and must be fully established prior to the treatment of participants. Before finalization, at least one DMC member, preferably the DMC chair, should review the protocol. This ensures that the DMC can provide input to sections related to and impacting the role and responsibility of the DMC. In addition, a DMC charter is required as part of the clinical trial application for some Health Authorities, which underscores the importance of an early DMC establishment.

At NN, two additional roles are assigned to tasks related to the DMC from the Global Safety Surveillance Department. The ‘DMC coordinator’ is responsible for handling the DMCs’ contracting and compliance with external healthcare professional (HCP) and healthcare organization (HCO) regulatory and financial aspects. The second role is a ‘DMC process manager’, who serves as the internal DMC subject matter expert and the go-to person for all DMC-related questions in general. These roles are critical for ensuring consistency and alignment across the various DMCs and as sponsor representatives, they must remain blinded and not have access to unblinded information.

Prior to the trial start date, a DMC kick-off meeting is held to initiate the collaboration between the DMC, sponsor and statistical vendor [3]. At this meeting, the trial protocol, the statistical plans, current knowledge of the product safety profile, and the DMC charter are presented and discussed with DMC members (Fig. 2).

The DMC charter is created based on a sponsor template document and includes a clear description of the roles and responsibilities of the DMC, the sponsor, and the statistical vendor. It documents rules of conduct for data review meetings, minimum data items to be reviewed by the DMC, and the process for providing recommendations to the sponsor. DMC feedback on the charter and on the interim analysis, if applicable, will be discussed at the DMC kick-off meeting. The DMC charter is approved and signed by the DMC chair and sponsor before the first patient’s treatment. Mock table shells for the DMC report should be prepared by the statistical vendor and will be agreed upon at the kick-off meeting. Any updates to table shells during trial conduct will only be discussed in closed meeting sessions to avoid any potential unblinding of the sponsor. Typically, timelines for the first data review meeting will be agreed upon at the kick-off meeting, and the communication line between the DMC, the statistical vendor, and the sponsor will be made clear to everyone.

## The Statistical Vendor and the Data Transfer Agreement

In parallel with the DMC establishment, the sponsor contracts the statistical vendor. Other common terms used for the independent statistical support team are the Statistical Data Analysis Center (SDAC) or Independent Statistical Reporting Group (ISRG) [16, 17]. This vendor and key staff shall, like the DMC members, confirm and declare to be independent of the sponsor and free of any financial or other COI as they have access to unblinded data. The statistical vendor prepares the data reports for the DMC and acts as a liaison between the DMC and the sponsor in relation to the data review meetings to ensure unblinded information is not inadvertently shared with the sponsor [16, 18].

The DMC relies heavily on the statistical and scientific quality of the DMC report, which necessitates a high level of statistical expertise from the vendor [19]. Therefore, selecting a vendor for the DMC involves several key considerations, including the vendor’s statistical capabilities, experience in the therapeutic area, and proven track record in supporting DMCs. Additionally, the vendor should possess regulatory knowledge and have robust systems and procedures to protect confidential data while ensuring compliance with relevant data protection regulations and Good Clinical Practice (GCP) standards.

At NN, we collaborate with several vendors to maintain the highest quality and efficiency of our projects. Costs, vendor collaboration, and communication skills are taken into account during this process. The DMC advisor at NN leads the vendor selection process, involving relevant stakeholders and considering these essential factors. Any feedback from the DMC or internal stakeholders regarding the collaboration or deliverables from the vendor is being addressed by NN on an ongoing basis, as well as at the end of the collaboration. This approach ensures that the DMC is continuously supported in the best possible way.

Prior to the first DMC data review meeting, a test data transfer is conducted from the sponsor to the statistical vendor. This step is key to ensuring that any operational data transfer issues are solved prior to the data transfer for the first data review meeting and that data is available to the statistical vendor for programming the data for the DMC reports. The statistical vendor receives raw data and is provided access to randomization codes to be able to unblind data as well as relevant information on mapping and data format.

At NN, a document called ‘responsibilities, timelines and logistic agreement’ (RTLA) defines internal stakeholder agreements regarding data extraction and delivery to the statistical vendor. It details timelines, data transfer instructions, and quality control for all stakeholders delivering data to the DMC. A unique RTLA is made for each DMC to ensure timely, high-quality data delivery and a consistent data transfer process. The DMC advisor is responsible for driving the RTLA preparation and coordinating inputs from key stakeholders such as data management, clinical operation, programmers, and event adjudication representatives, if applicable. The DMC advisor ensures communication of data transfer timelines to all stakeholders.

The statistical vendor must utilize a highly secured file-sharing system to enable the sponsor to upload confidential data files and provide the DMC with separate, controlled access to all data reports, protocols, and other relevant documents.

## DMC Data Review Meetings

The DMC charter defines the expected frequency of DMC meetings, typically occurring 2–4 times per year. The DMC advisor coordinates and plans the DMC data review meetings preferably with a 12-month notice. In addition, the DMC advisor organizes a pre-meeting with NN key stakeholders to agree upon the agenda and pre-read any documents to be shared with the DMC prior to the DMC meeting. Common agenda topics include details on the patient recruitment/retention status or other important trial or safety-related information supporting the data evaluation by the DMC.

The DMC meeting begins with an open session attended by the DMC, the statistical vendor, and NN stakeholders (Fig. 2). NN presents the pre-read detailed topics, and questions or clarifications required by the DMC are addressed. The discussion in the open sessions is restricted to blinded data. Following the open session, the closed session is initiated, during which the DMC and statistical vendor discuss unblinded data. Based on the data evaluation, the DMC recommends NN on whether to continue, modify or terminate the trial. The recommendation is provided to NN at the subsequent open session, which occurs immediately after the closed session where any potential follow-up actions for the next meeting are also provided. NN has a set 24-hour reporting timeframe for the DMC chair to provide the signed DMC recommendation, ensuring timely submission to health authorities and international ethics committees/institutional review boards according to local requirements.

If the DMC recommends continuing the trial without changes, the ‘qualified person for pharmacovigilance’ (QPPV) will be informed immediately after the DMC data review meeting through a written update, whereas the NN safety committee will be informed at the next upcoming meeting. If the DMC recommends modifying the trial, further actions will depend on the specific recommendation. If the recommendation is minor logistical changes, this will normally be implemented by the trial team. However, if the suggested modification is major and due to a safety concern, or if the recommendation is to terminate the trial, the QPPV will immediately communicate with either the DMC or the DMC chair to understand the concern and evaluate whether actions are needed for the compound in question outside of the specific trial, such as in other ongoing trials with the same compound. As the sponsor of the trial, the NN safety committee holds the final responsibility for deciding on any actions based on the DMC’s recommendation, including a potential need for unblinding data. Timely communication with the Health Authorities will be initiated as applicable, and if there is a Steering Committee, it will also be consulted.

In situations where a DMC is responsible for an interim efficacy analysis, a fourth recommendation has been introduced in addition to the three traditional recommendations: continue, modify, or terminate the trial. This additional recommendation, “to close due to the sponsor’s pre-specified efficacy interim evaluation,” is also sent to Health Authorities. By explicitly stating in the recommendation that the closure is due to efficacy, there is no risk that Health Authorities will misunderstand the recommendation as a safety issue. If the DMC recommends closing the trial for efficacy, a company announcement is also prepared and ready to be communicated.

At NN, DMC meetings are now primarily held virtually. This shift is a result of improvements in video-communication platform utilization and was further facilitated by the increased need during the COVID-19 pandemic. Although the use of online video-communication platforms offers benefits, such as increased flexibility for attendance in a busy work schedule and benefits for climate in terms of reduced CO_2_ emissions from travels, NN encourages in-person DMC kick-off and final DMC meetings. In-person meetings help foster close working relationships and robust collaboration between the DMC members, the statistical vendor, and NN. At the final meeting, DMC members are encouraged to provide feedback on their collaboration with NN. This input is valuable to NN as a sponsor, ensuring the robustness and consistency of the process across DMCs.

## Quality Control and Business Ethics

A quality management system must be in place to ensure processes are in accordance with regulatory guidelines. The DMC process manager reviews new regulatory requirements and ensures that standard operating processes are updated and in compliance. Furthermore, processes are continuously improved based on regular internal audits and Health Authority inspections.

Over the last decades, there has been increasing focus on the interaction of the pharmaceutical industry with HCPs and HCOs, and strict rules have been implemented regarding working relationships and business ethics. As such, all NN employees working with HCPs are required to recurrently complete business ethics training and utilize internally developed tools and apps to support adherence to country-specific regulatory requirements. Should a challenging compliance issue arise, a dedicated global business ethics department or the local affiliate handling business ethics/compliance can be consulted.

## Discussion

This article was motivated by positive feedback from former members serving at a DMC for a NN trial. Patient safety is NN’s key priority, and a strong DMC is integral to achieving this objective. This publication outlines the processes employed at NN to ensure effective and high-performing DMCs, while also highlighting some of the challenges and considerations encountered.

One notable challenge is the identification and contracting of DMC members. This process needs to be initiated early, as it is often time-consuming to find suitable DMC candidates with the right qualifications and without any COI. The assessment of DMC members’ independence is of high priority to NN to allow for an unbiased evaluation of the study data. Currently, there are no clear definitions of what is considered a COI in the regulatory guidelines and recommendations [20–22]. As such, the interpretation of COI remains subjective and requires detailed discussion and assessment by the sponsor.

Some DMC members consider NN to be conservative regarding the requirements and assessments for transparency. It is important to clarify the rationale for robust and strict assessment of COI to the DMC members and to provide guidance on what is considered acceptable and non-acceptable. Identifying members with the required expertise without conflicting activities can be challenging. There have been situations where changes in a DMC member’s work-related activities and responsibilities have been considered a COI by NN, and the DMC member was asked to choose between taking on the DMC role or continuing their involvement in the other activity. In some cases, NN has had to terminate the contract for serving on an NN DMC and consequently immediately replace the member to maintain an operational DMC. This is, of course, very unfortunate but rarely happens. Ideally, DMC members are not replaced throughout the entire trial conduct to ensure continuity.

Sponsors managing multiple DMCs must maintain a comprehensive overview of rules and legislations related to HCP engagements to ensure compliance with country-specific and global regulatory requirements. Examples of country-specific requirements include pre-notification to health authorities well in advance of meetings, requirements for specific hospital/university work permissions, and strict thresholds and caps on HCP honoraria. This adds to the complexity and can be challenging when setting up and managing DMCs.

To ensure the best possible quality and up-to-date dataset for the DMC reports [16, 18, 23], a strong collaboration is required between the different stakeholders involved in the delivery of data. It is a team effort across various functional areas to ensure that the data is complete and cleaned to the extent possible prior to transfer to the statistical vendor. Timely communication of data transfer dates to all stakeholders enables the team to handle any unforeseen data issues before delivery to the statistical vendor. For data accuracy and completeness, it is also recommended to align the planned regular data cleaning cycles as closely as possible to the data transfer dates. The DMC usually receives the data report one week prior to the meeting, and the data cutoff date for the report is balanced between the need for up-to-date data and the time required to prepare the report.

A close and trustful collaboration between the statistical vendor and sponsor is crucial to ensure that the DMC receives timely and accurate data, and that the statistical vendor is well-equipped to support the DMC. It is important that the DMC members feel confident that the data they review is of the best possible quality and that any questions can be addressed by the statistical vendor, if related to unblinded data, during the closed part of the meeting or by NN during the open part of the meeting. This requires that the relevant people attend the open meeting and are well-prepared to discuss perspectives of quality of the data and address other questions raised by the DMC to aid the discussions and assessment of data during the closed meeting.

## Conclusion

In conclusion, the dynamic, strictly regulated environment of clinical drug development and DMCs mandates a robust process and quality management system. Furthermore, strong collaboration between the sponsor’s internal stakeholders and the external statistical vendor is crucial to support the work of the DMC. This ensures the effective and efficient performance of the DMC and, ultimately, the safety of the patients in the clinical trials. By sharing our insights and practices, we hope this publication can serve as an inspiration for other companies in their handling of DMCs.

## Data Availability

No datasets were generated or analysed during the current study.
